# Curcumin Prevents High Fat Diet Induced Insulin Resistance and Obesity via Attenuating Lipogenesis in Liver and Inflammatory Pathway in Adipocytes

**DOI:** 10.1371/journal.pone.0028784

**Published:** 2012-01-09

**Authors:** Weijuan Shao, Zhiwen Yu, Yuting Chiang, Yi Yang, Tuanyao Chai, Warren Foltz, Huogen Lu, I. George Fantus, Tianru Jin

**Affiliations:** 1 Division of Cell and Molecular Biology, Toronto General Research Institute, University Health Network, Toronto, Canada; 2 Department of Nutrition, Public Health Institute, Sun Yat-Sen University, Guangzhou, China; 3 Department of Physiology, University of Toronto, Toronto, Canada; 4 Radiation Medicine Program, The STTARR Innovation Centre, Princess Margaret Hospital, University Health Network, Toronto, Canada; 5 Banting and Best Diabetes Centre, Faculty of Medicine, University of Toronto, Toronto, Canada; Erlangen University, Germany

## Abstract

**Background:**

Mechanisms underlying the attenuation of body weight gain and insulin resistance in response to high fat diet (HFD) by the curry compound curcumin need to be further explored. Although the attenuation of the inflammatory pathway is an accepted mechanism, a recent study suggested that curcumin stimulates Wnt signaling pathway and hence suppresses adipogenic differentiation. This is in contrast with the known repressive effect of curcumin on Wnt signaling in other cell lineages.

**Methodology and Principal Findings:**

We conducted the examination on low fat diet, or HFD fed C57BL/6J mice with or without curcumin intervention for 28 weeks. Curcumin significantly attenuated the effect of HFD on glucose disposal, body weight/fat gain, as well as the development of insulin resistance. No stimulatory effect on Wnt activation was observed in the mature fat tissue. In addition, curcumin did not stimulate Wnt signaling in vitro in primary rat adipocytes. Furthermore, curcumin inhibited lipogenic gene expression in the liver and blocked the effects of HFD on macrophage infiltration and the inflammatory pathway in the adipose tissue.

**Conclusions and Significance:**

We conclude that the beneficial effect of curcumin during HFD consumption is mediated by attenuating lipogenic gene expression in the liver and the inflammatory response in the adipose tissue, in the absence of stimulation of Wnt signaling in mature adipocytes.

## Introduction

Type 2 diabetes mellitus (T2D) is increasing at an alarming rate in both developed and developing countries, associated with a combined health and economic burden worldwide [1]. The epidemic of obesity and its related insulin resistance have contributed significantly to the incidence of diabetes. It is now generally accepted that both obesity and T2D are associated with low grade chronic inflammation and that adipose tissue appears to be the first organ that is affected [2]. The development of inflammation and oxidative stress in adipose tissue leads to insulin resistance [3], [4]. Furthermore, accelerated hepatic lipogenic gene expression and reduced liver fat export may also contribute to the development of obesity [5], [6].

Many naturally occurring dietary polyphenols possess antioxidant and anti-inflammatory properties [7]. This could be achieved by modulating an inflammatory or oxidative signaling pathway, including NF-κB, Nrf2, and/or MAPK-dependent signaling pathways [7], [8], [9]. Certain dietary polyphenols, such as curcumin, also possess the anti-carcinogenic effects. One potential mechanism of curcumin to repress tumorigenesis has been suggested to be the inhibition of Wnt signaling, an essential pathway for embryogenesis and cell proliferation [10], [11].

Curcumin, a low-molecular-weight polyphenol derived from the herbal remedy and dietary spice turmeric, was found to prevent obesity and diabetes in mouse models [12]. Mechanistically, curcumin may exert its beneficial effects via reducing insulin and leptin resistance, attenuating inflammatory cytokine expression, accelerating fatty acid oxidation, as well as increasing antioxidant enzyme expression [7]. In addition, curcumin could also function as an inhibitor of p300 histone acetyltransferase (HAT), a potential molecular mechanism for cancer prevention and cardiovascular improvement [13], [14].

The Wnt/β-catenin (β-cat) signaling pathway was initially discovered in colon cancer and in developmental studies of *Drosophila* and frogs [15]. The role of the canonical Wnt signaling pathway (defined as Wnt pathway hereafter) in metabolic homeostasis has recently received increasing attention [15], [16], [17]. Activation of Wnt pathway increases cellular and nuclear β-cat level, which represses adipogenesis, while the inhibition of Wnt signaling is required for PPARγ induction and preadipocyte differentiation [18]. A very recent study showed that curcumin stimulates Wnt/β-cat signaling in 3T3-L1 preadipocytes and hence suppresses adipogenic differentiation [19]. Although this finding provides a potential molecular mechanism for the effect of curcumin in attenuating obesity, it is contradictory with other reports in two ways. First, numerous studies have indicated that curcumin exerts its anti-cancer effect via repressing Wnt signaling [10], [11]. Second, Wnt activation in mature adipocytes was shown to induce insulin resistance [20], while curcumin is known to attenuate insulin resistance [12].

In this study we have examined the effect of dietary curcumin in a HFD mouse model in which the development of obesity and insulin insensitivity was relatively slow due to the administration of 45% rather than 60% of calories from fat [8]. In this mouse model as well as in primary rat adipocytes, we did not observe stimulation of curcumin on Wnt pathway components or Wnt target gene expression. However, curcumin attenuated lipogenic gene expression in hepatocytes, and blocked the effect of HFD on the inflammatory response in the adipose tissue, associated with decreased weight/fat gain, and the maintenance of normal glucose tolerance and insulin sensitivity.

## Materials and Methods

### Materials

Curcumin was purchased from Sigma (St. Louis, MO). Antibodies against PKB/Akt, phosphorylated PKB (Ser473), phosphorylated β-cat (Ser675 β-cat), GSK-3β, phosphorylated GSK-3α/β, and β-actin were obtained from Cell Signaling Technology (Beverly, MA). Antibodies against NF-κB, total β-cat, c-Myc, cyclin D1, phosphorylated JNK, SREBP1-c, F4/80 and HO-1 were from Santa Cruz Biotechnology (Santa Cruz, CA). Thioredoxin-interacting protein (TxNIP) antibody was obtained from MBL International Corporation (Woburn, MA). ChREBP antibody was purchased from Novus Biologicals, (Littleton, CO). Kits for glucose, cholesterol, free fatty acids (FFA), and HDL assessment were from Abcam (Cambridge, MA). Triglyceride (TG) assay kit was from Cayman Chemical (Ann Arbor, Michigan). Leptin/insulin ELISA kit were from Crystal Chem. Inc. (Downers Grove, IL). Adiponectin ELISA kit was from R&D Systems (Minneapolis, MN). GSH/GSSG assay kit was from Oxford Biomedical Research (Oxford, MI) and the method for determining plasma GSH/GSSG ratio was described previously [8].

### Animal care and treatment

Male C57BL/6J mice from Jackson Laboratory (Bar Harbor, Maine) were housed 5 per cage under the conditions of constant temperature (22°C), a light/dark cycle of 12 h with free access to food and water. Thirty-six five-week-old mice were randomly divided into three groups. Group A were fed with the low-fat-diet (LFD, control diet, 10% Kcal from fat), while group B with the HFD (45% Kcal from soy bean fat). Group C were fed with HFD with curcumin (4g/kg diet) added 2 days/week (Mondays and Thursdays). Diets were prepared by Harlan Tekland (Madison, WI) [8]. The animal experiments and protocols were approved by the University Health Network Animal Care Committee and performed in accordance with the guidelines of the Canadian Council of Animal Care. The approval ID for this study is AUP1561.11.

### MRI assessment of total fat mass and lipid content

MRI was performed using a 7 tesla Biospec 70/30 USR (Bruker BioSpin MRI GmbH, Ettlingen, Germany) in The STTARR Innovation Centre, Radiation Medicine Program, Princess Margaret Hospital, University Health Network, Toronto, Canada, as previously described [8].

### Intraperitoneal (i.p.) glucose, insulin and pyruvate tolerance tests

Mice were fasted overnight for glucose tolerance tests; or fasted for 6 h for insulin and pyruvate tolerance tests. Following the fasting, glucose (2 g/kg), insulin (0.65 U/kg) or pyruvate (2 g/kg) was i.p. injected. Blood samples collected from tail vein were used for glucose measurements.

### Determination of blood biochemistry and liver TG content

Ambient levels of plasma glucose, TG, total cholesterol, FFA and HDL after an overnight fast were measured using kits following the manufacturers' instructions. Liver TG content was determined as described [21] .

### Quantitative real-time RT-PCR

Real-time PCR was performed using iQ™ Sybr Green (Bio-Rad, Mississauga, On., Canada) using the Rotorgene according to the protocols provided by the manufacturer. The relative mRNA transcript levels were calculated according to the 2^−ΔΔCt^ method. Oligonucleotide primers for RT-PCR are summarized in Table 1.

**Table 1:** List of oligonucleotide primers utilized in this study.

	Genbank#	DNA Sequence	Anticipated size of the product (bp)
ChREBP	NM_021455.3	Forward:5′-ACCGGGGTGCCCATCACACA-3′	315
		Reverse: 5′-CTGCCCGTGTGGCTTGCTCA-3′	
SREBP-1c	NM_011480.2	Forward:5′-TAGAGCATATCCCCCAGGTG-3′	244
		Reverse:5′-GGTACGGGCCACAAGAAGTA-3′	
L-PK	NM_013631.1	Forward:5′-GAGTCGGAGGTGGAAATTGT-3′	173
		Reverse:5′-CCGCACCACTAAGGAGATGA-3′	
TxNIP	NM_023719.2	Forward:5′-AGAGCAGCCTACAGGTGAGA-3′	260
		Reverse:5′-TCTCCTTTTTGGCAGACACT-3′	

### Cell lines and primary cell cultures

HepG2 cells were purchased from ATCC (Manassas, VA). They were cultured in α-MEM supplemented with 10% fetal bovine serum (FBS). Epididymal fat pads from male wild-type Wistar rats fed a normal diet were excised, minced in DMEM containing 3% BSA and digested with 1 mg/ml collagenase Type 1 (Worthington Biochemical Corporation, Lakewood, NJ) at 37°C for 60 min. The digested fat tissue was filtered through a mesh (100 µm) and centrifuged at 500 rpm for 5 min to separate floating adipocytes from the medium**.** After washing 4 times, cells were maintained in DMEM containing 1% BSA at 37°C, with additions as indicated.

### Western blot analysis

Tissue samples were homogenized and equal amounts of proteins (30 µg) were separated with denaturing SDS 10% polyacrylamide gels. The method for Western blotting was as previously described [8], [22].

### Luciferase (LUC) reporter analysis

The generation of ChREBP promoter constructs, as well as the method for LUC reporter gene analysis was described previously [23], [24].

### Immuno and Histological staining

The sections of epididymal adipose tissue were fixed in 10% formalin, dehydrated, and embedded in paraffin. Adipose tissue sections were stained with hematoxylin and eosin (H&E) to examine the morphology and with the F4/80 antibody to detect macrophages. Hepatic Oil O Red staining was conducted with the routine method.

### Statistics

All results are expressed as mean ± SEM. Statistical significance was assessed by ANOVA. *P* value less than 0.05 was considered to be statistically significant.

## Results

### Long term dietary curcumin administration prevented HFD-induced body-weight gain and obesity

**Figure 1:**
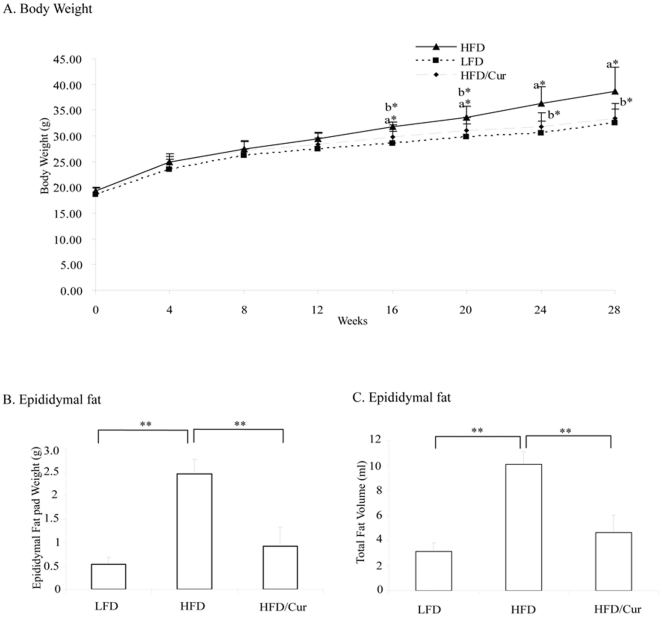
Long term dietary curcumin supplementation prevents HFD-induced obesity and fat mass. (**A**) Comparison of the body weight change of mice fed with LFD, HFD or HFD plus curcumin for 28 weeks (n = 4 for LFD or HFD, and n = 8 for HFD/curcumin). a, LFD versus HFD; b, HFD versus HFD/curcumin. *, p<0.05. (**B**) Weights of the epididymal fat pad (n = 4 for LFD and HFD, n = 8 for HFD/curcumin). **, p<0.01. (**C**) Total body fat volume assessed by MRI at 26 weeks (n = 4 for all three groups).

In this chronic HFD mouse model, body-weight increased significantly only after 16 weeks of HFD feeding (Fig. 1A). From this time point to the end of the 28th week, dietary curcumin significantly blocked the effect of HFD on body-weight gain (Fig. 1A). HFD also significantly increased the weight of epididymal fat pads, while curcumin supplementation significantly blocked this stimulation (Fig. 1B). We then assessed total fat mass of the animals by MRI and found stimulation by HFD and inhibition by curcumin supplementation (Fig. 1C).

HFD feeding increased the fasting plasma insulin level (0.28±0.15 ng/ml for LFD, 0.67±0.10 ng/ml for HFD, p<0.05), while dietary curcumin resulted a reduction on its level (0.10±0.06 ng/ml in HFD plus curcumin, p<0.01 versus HFD). Although HFD reduced and curcumin increased plasma adiponectin level, the differences did not reach statistical significance (7.26±1.08 µg/ml for LFD, 5.99±2.14 µg/ml for HFD, and 7.22±1.09 µg/ml for HFD/curcumin).

### Curcumin improved glucose disposal and insulin sensitivity

**Figure 2:**
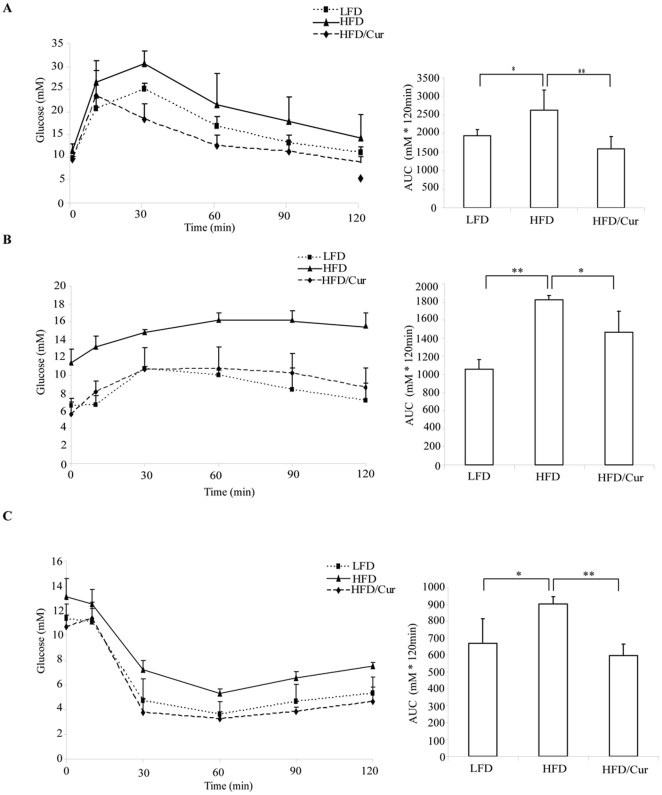
Curcumin supplementation improves glucose disposal and insulin sensitivity. Assessment of glucose metabolism in animals with LFD, HFD or HFD plus curcumin feeding. (**A**) IPGTT (n = 4, 20 weeks), (**B**) IPPTT **(**n = 4, 23 weeks), and (**C**) IPITT (n = 4, 26 weeks). AUC, area under the curve. *, p<0.05; **, p<0.01.

To evaluate the functional outcome of long-term curcumin supplementation on glucose homeostasis, we conducted an intraperitoneal glucose tolerance test (IPGTT). A representative IPGTT result performed at the end of the 20th week is presented in Fig. 2A. Blood glucose levels were higher in the HFD animals while curcumin significantly blocked these increase. To determine whether liver was implicated in the improvement of glucose disposal by curcumin, we conducted intraperitoneal pyruvate tolerance tests (IPPTT) at the end of the 23^rd^ week. As shown in Fig. 2B, glucose production following pyruvate administration was significantly enhanced in the HFD fed mice, while curcumin significantly blocked the effect of HFD, indicating that increased hepatic gluconeogenesis by HFD feeding was inhibited by dietary curcumin. Finally, we conducted intraperitoneal insulin tolerance tests (IPITT) at the end of 26 weeks. Insulin was less effective in lowing glucose level in HFD animals, while curcumin supplementation efficiently blocked this effect of HFD. These data suggest that curcumin improves whole body glucose disposal by both stimulation of insulin sensitivity and inhibition of hepatic gluconeogenesis.

### Curcumin improved insulin signaling in adipose tissue and hepatocytes

**Figure 3:**
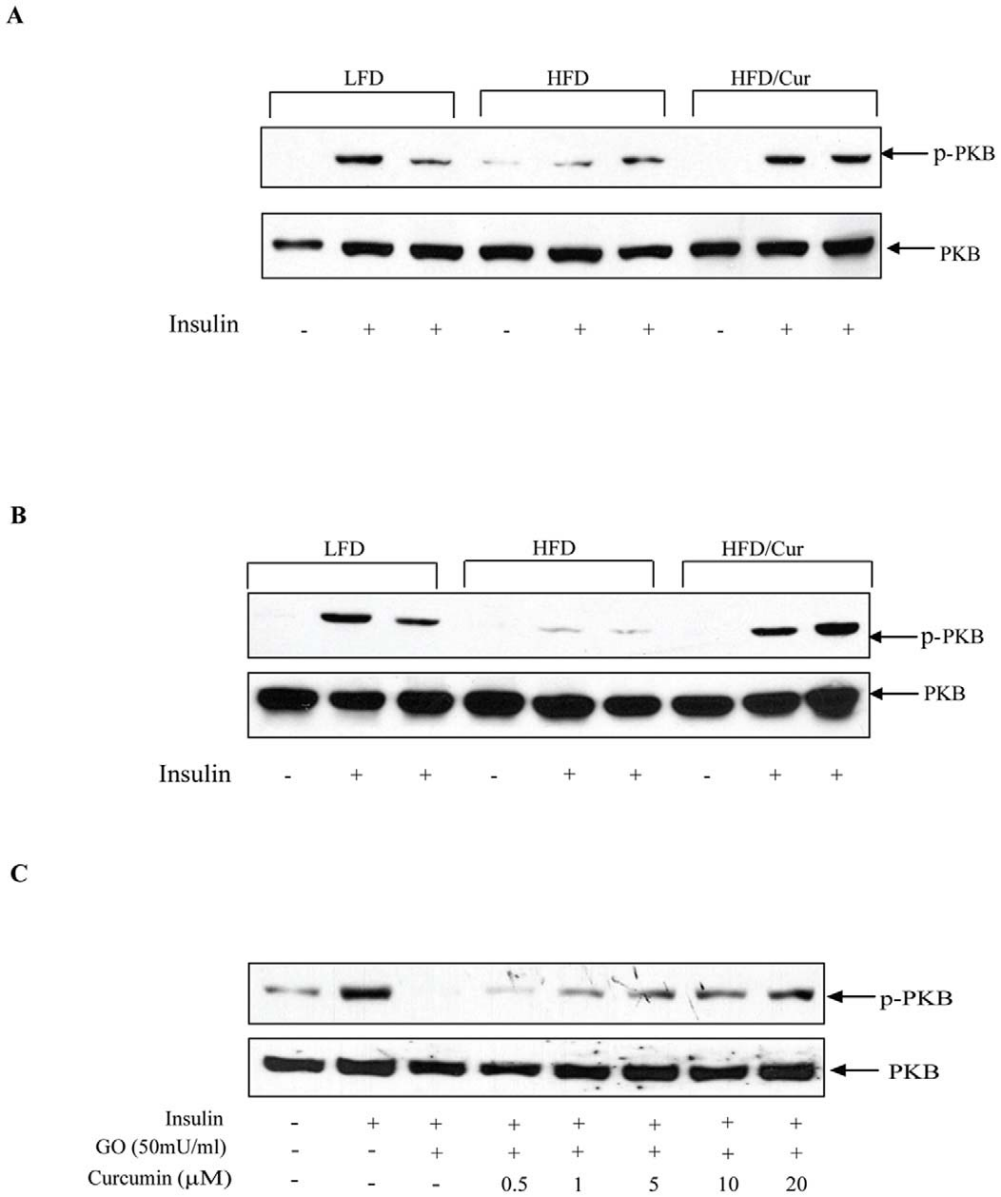
Curcumin improves insulin stimulated PKB phosphorylation in fat tissue and hepatocytes. (**A**) and (**B**) The three groups of mice fed with the diets as indicated for 28 weeks were fasted overnight and injected with PBS or insulin. After 30 min, the mice were sacrificed. Samples of epididymal fat pad (**A**) and liver (**B**) were prepared and immunoblotted with PKB or Ser473 phosphorylated PKB (p-PKB) antibody. (**C**) HepG2 cells were pre-treated with or without curcumin at indicated concentration overnight, followed by a 6 h treatment with or without glucose oxidase (GO). The cells were then further treated with or without insulin (100 nM) for 10 min, followed by PKB and p-PKB immunoblotting. The blots shown are representative of 3 separated experiments.

To further explore molecular mechanisms underlying the protective effects of dietary curcumin, we assessed insulin signaling by determining PKB/Akt Ser473 phosphorylation in response to insulin injection in insulin responsive tissues. We did not see the deleterious effect of our HFD on insulin stimulated PKB Ser473 phosphorylation in soleus and gastrocnemius muscles (Figure S1). However, in both adipose tissue and liver, HFD impaired insulin-stimulated PKB phosphorylation and the impairment was prevented by curcumin supplementation (Fig. 3A and 3B). Fig. 3C shows that in the human hepatic cell line HepG2, insulin stimulated PKB Ser473 phosphorylation (lanes 1 and 2), while glucose oxidase (GO) pre-treatment, which is known to induce oxidative stress [25], blocked the stimulatory effect of insulin (lane 3). Curcumin, however, was found to dose-dependently restore the stimulatory effect of insulin, in the presence of GO (lanes 4–8).

### Curcumin did not stimulate Wnt signaling in mature adipocytes

**Figure 4:**
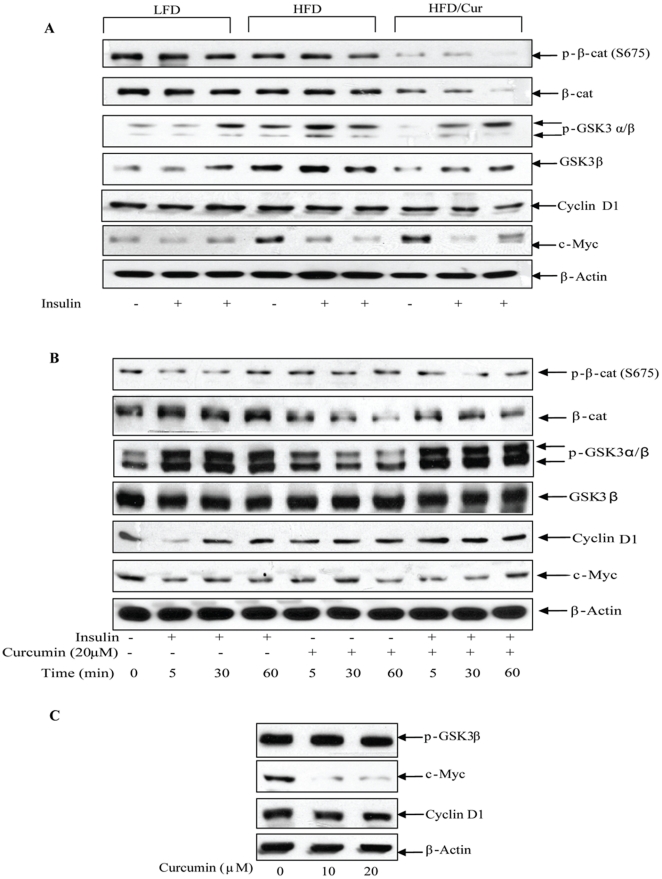
Curcumin does not stimulate the Wnt signaling pathway in mature adipocytes. (**A**) Samples from epididymal fat pad of the three groups of mice were prepared for Western blotting. β-cat and GSK-3 represent Wnt pathway effectors, while cyclin D1 and c-Myc are two known Wnt target genes. (**B**) Rat primary adipocytes were prepared and treated with insulin (100 nM), or curcumin (20 µM), or insulin plus curcumin for 0, 5, 30, and 60 min. Samples were collected for Western blotting, with indicated antibody. (**C**) Rat primary adipocytes were prepared and treated with 10 or 20 µM curcumin for 4 h, followed by Western blotting with indicated antibody. All panels show the representative blot (n = 3).

We have observed previously that insulin stimulates β-cat Ser675 phosphorylation in a gut endocrine L cell line (data not shown) and non-endocrine intestinal cells [22], which represents a novel mechanism for the crosstalk between insulin (possibly other signaling molecules) and the Wnt signaling pathway [26], [27]. Here we tested whether this crosstalk occurs in mature adipocytes. After the mice received intraperitoneal insulin injection for 30 min, we took the epididymal fat tissue for Western blotting. As shown in Fig. 4A, in adipose tissue, there was no stimulation on β-cat Ser675 phosphorylation in any of the three groups of animals, although stimulation of GSK3α/β phosphorylation was appreciable. Furthermore, curcumin reduced the overall levels of both Ser675 β-cat and total β-cat (Fig. 4A). In addition, HFD increased total GSK-3β level, while curcumin reduced the level of total GSK-3β. Finally, curcumin supplementation showed no stimulation on the expression of c-Myc and cyclin D1 protein, two downstream targets of the Wnt signaling pathway (Fig. 4A) [28]. Real time RT-PCR also showed that dietary curcumin did not stimulate the expression of cyclin D1 and LRP5 mRNA (data not shown). Taken together, these observations indicate that in this animal model, curcumin did not stimulate Wnt pathway components or Wnt pathway downstream target genes in mature adipocytes.

We then conducted further experiments in primary rat adipocytes. Fig. 4B shows that treating rat adipocytes with insulin, or curcumin, or insulin plus curcumin for 5 to 60 min generated no significant effect on total GSK-3β or Ser675 β-cat expression level. Total β-cat level, however, appeared to be repressed by curcumin treatment. Furthermore, curcumin did not block the stimulatory effect of insulin on GSK-3 phosphorylation. Within 60 min, curcumin or insulin had no observable effect on the expression of c-Myc or cyclin D1. We then extended the treatment time by curcumin to 4 h. As shown in Fig. 4C, curcumin moderately repressed the expression cyclin D1 and greatly repressed the expression of c-Myc, associated with no appreciable effect on GSK-3 phosphorylation. Taken together, we did not see a stimulation of curcumin on Wnt pathway components or Wnt target gene expression in vivo in the cultured mature adipocytes.

### Curcumin attenuated the inflammatory and oxidative pathway in adipocytes

**Figure 5:**
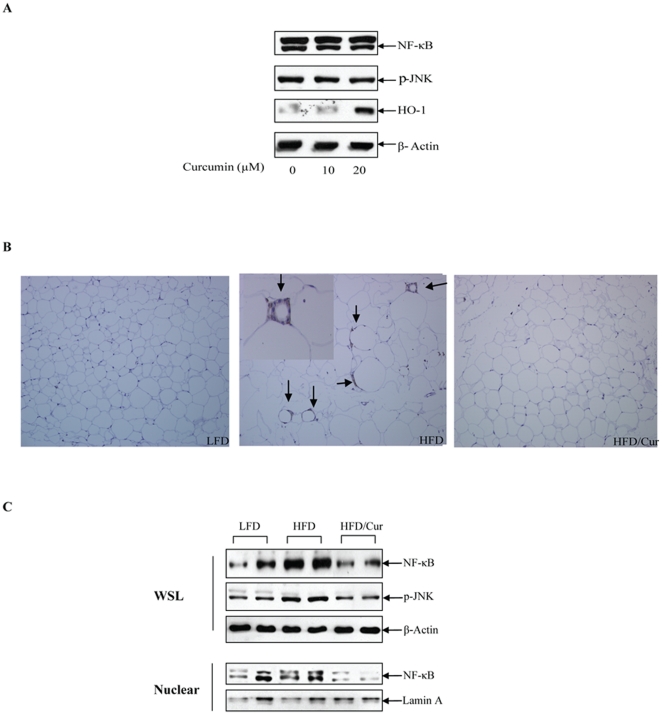
Curcumin increases HO-1 expression and reduces inflammatory markers in mature adipocytes. (**A**) Rat primary adipocytes were prepared and treated with 10 or 20 µM curcumin for 4 h. Western blotting was performed with the indicated antibodies. (**B**) Immunostaining was performed for the detection of macrophage infiltration marker F4/80 in the fat tissue of the three groups of mice. Arrows show the positive staining. No macrophage infiltration was observed in LFD or HFD/Curcumin animals. (**C**) Samples from epididymal fat pad of the three groups of mice were prepared for Western blotting. WSL, whole cell lysates; Nuclear, nuclear extracts. Panel A and C show representative blots (n = 3).

In mature adipocytes, increased oxidative stress in response to HFD could contribute to increased inflammatory signaling, which is at least partially responsible for the impairment of whole body insulin sensitivity [29]. Curcumin was found to attenuate oxidative stress and reduce the inflammatory response [7]. We observed that HFD reduced the ratio of GSH/GSSG (1.23±0.27 for LFD, compared with 0.9±0.17 for HFD), but the difference did not reach statistical significance. Curcumin supplementation, however, significantly increased GSH/GSSG ratio (1.39±0.24), when compared with the HFD group (p<0.01). In addition, we found that in rat primary adipocytes, 20 µM curcumin significantly increased the expression of HO-1, a fundamentally important enzyme of the endogenous anti-oxidant system (Fig. 5A). Curcumin treatment, however, did not generate a substantial effect on NF κB or pJNK levels (Fig. 5A), possibly due to the absence of an oxidative stress in this in vitro assay. Furthermore, dietary curcumin not only attenuated the stimulatory effect of HFD on the size of adipocytes, but also completely blocked the effect of HFD on macrophage infiltration, determined by the expression of F4/80 in epididymal adipocytes (Fig. 5B). Finally, in mice, curcumin attenuated the stimulation of HFD on the inflammatory response, indicated by the inhibition of the rise in NF-κB expression level (both in whole cell lysates and nuclear extract) and JNK signaling pathway activation (Fig. 5C). These observations are generally consistent with previous findings by other groups [12], [30], and collectively suggest that the inhibition of oxidative stress and the inflammatory pathway in adipose tissue are among the mechanisms underlying the protective effect of dietary curcumin in improving insulin signaling, attenuating obesity, and preventing the development of diabetes.

### Curcumin reduced hepatic lipogenic gene expression

**Figure 6:**
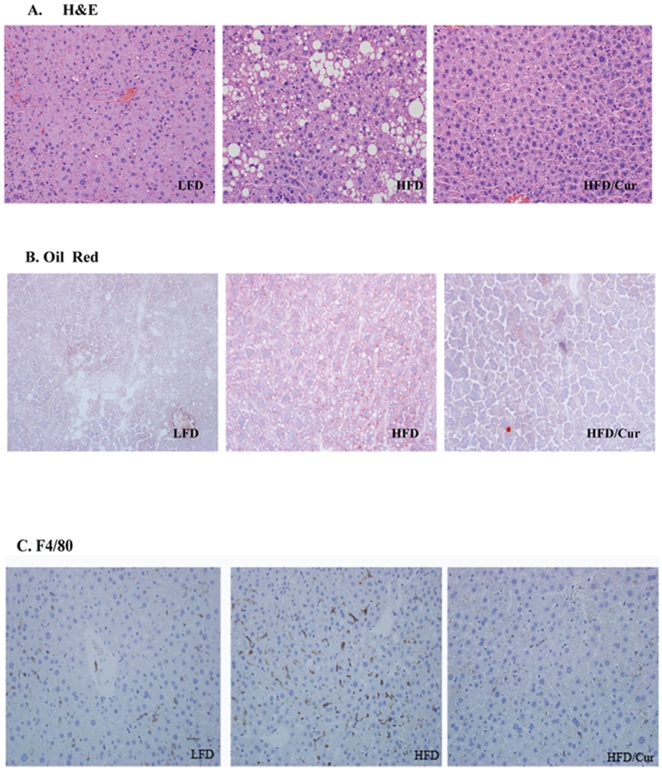
Histological assessment of mouse liver. Representative histological slides show lipid content (**A**, H&E staining; **B**, Oil Red O staining**)** and macrophage infiltration (**C**) in the three groups of mice.

We have then examined histological changes in the liver of each group of the mice. As shown in Fig. 6, HFD consumption increased liver lipid content, demonstrated by both H&E staining (Fig. 6A) and Oil Red O staining (Fig. 6B). In the curcumin group, however, the effect of HFD on the elevation of lipid content was blocked. The effect of HFD on macrophage infiltration (assessed by F4/80 staining) was also blocked by curcumin consumption (Fig. 6C).

**Figure 7:**
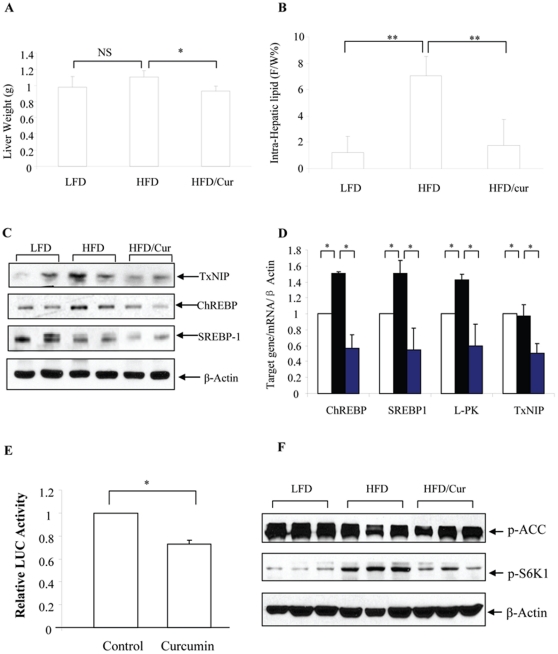
Curcumin reduces intra-hepatic lipid content and lipogenic gene expression. (**A**) Curcumin supplementation reduced liver weight in HFD fed mice (n = 4 for all 3 groups). (**B**) MRI shows that curcumin supplementation reduced intra-hepatic lipid content (n = 4 for all 3 groups). (**C**) Curcumin supplementation reduced the expression of TxNIP, ChREBP and SREBP-1c in liver of HFD fed mice (A representative blot, n = 3). (**D**) Curcumin supplementation reduced liver ChREBP, SREBP-1c, L-PK and TxNIP mRNA expression in HFD fed mice (n = 3). (**E**) The ChREBP-LUC reporter plasmid construct (3 µg) were transfected into HepG2 cells for 24 h, followed by a 20 h serum starvation and a 4 h curcumin (20 µM) treatment. The data are presented as mean ± SEM (n = 3). *; p<0.05; **, p<0.01. F. Curcumin supplementation blocked the stimulatory effect of HFD on S6K1 phosphorylation in the liver.

In this chronic HFD mouse model, although liver weight was not significantly increased (Fig. 7A), intra-hepatic lipid content (assessed by MRI) was increased more than 6 fold (Fig. 7B), consistent with the observation by H&E and Oil Red O staining. Curcumin significantly reduced liver weight (Fig. 7A) and blocked the effect of HFD on increasing intra-hepatic lipid content (Fig. 7B). Furthermore, curcumin reduced hepatic NF-κB level, although our HFD did not cause the increase of hepatic NF-κB level (Figure S2).

ChREBP and SREBP1-c are two well known transcription factors which stimulate lipogenic gene expression, while L-PK is a downstream target of ChREBP [31]. We found that in this HFD mouse model, hepatic ChREBP protein level but not SREBP-1c protein level, was increased (Fig. 7C). However, dietary curcumin reduced the levels of these two transcription factors (Fig. 7C). At mRNA level, dietary curcumin significantly reduced the amounts of SREBP-1c, ChREBP and L-PK (Fig. 7D). The mRNA level of Stearoyl-coenzyme A desaturase 1 (SCD1) in our HFD mice, however, was not increased. Curcumin treatment did not generate a significant change on its expression (Figure S3).

TxNIP is a sensor of glucose and oxidative phosphorylation status [32]. A recent study indicated that TxNIP transcription can be stimulated by ChREBP [33]. We found that TxNIP protein level in the liver was higher in the animals of the HFD group, while curcumin supplementation reduced its level (Fig. 7C). Curcumin supplementation also reduced TxNIP mRNA level (Fig. 7D). Furthermore, we conducted LUC reporter analysis, showing that in the HepG2 cell line, ChREBP promoter activity was repressed by curcumin treatment (Fig. 7E). Finally, we found that HFD feeding increased the phosphorylation of S6K1, a downstream target of mTOR signaling (Fig. 7F).

## Discussion

Curcumin is the principal curcuminoid of the popular spice turmeric utilized in Indian and other South Asia countries, which is a member of the ginger family. This plant polyphenolic compound has anti-tumor, anti-proliferative, anti-oxidant, and anti-inflammatory properties [7]. Since the last decade, a few clinical trials have been conducted, showing the therapeutic effects of curcumin on various cancers and Alzheimer's disease [34].

In C57BL/6J HFD fed mice, oral curcumin supplementation was shown to prevent the development of obesity-associated inflammation, insulin resistance, as well as diabetes [12]. The beneficial effect of curcumin in that study was mainly attributed to the reduction of macrophage infiltration of the adipose tissue, the increase of adiponectin production, as well as the decrease of hepatic NF-κB activity [7], [12]. The anti-adipogenic effect of curcumin was then demonstrated in the 3T3-L1 cell model by other groups [35], [36]. The stimulation of HFD on hepatic NF-κB level, however, was not observed in the current study with our chronic HFD model, although curcumin supplementation decreased NF-κB level in the liver (Figure S2).

Insulin resistance and obesity in C57BL/6J mice are often induced in the short-term by feeding a diet containing saturated fatty acids (45%) or with a mixed fatty acid diet with 60% energy from fat. In the current study we utilized a chronic HFD feeding model, in which mice did not develop obesity before 16 weeks, and the deleterious effect of HFD on both the morphology of the liver and plasma metabolic profiles were not as severe as the utilization of regular HFD [8] (data not shown). As presented, although our HFD reduced plasma adiponectin level, it did not reach statistical significance. Nevertheless, curcumin consumption generated a significant increase of plasma adiponectin. Furthermore, instead of routinely providing curcumin-containing HFD with every meal [12], we provided the curcumin-supplemented diet only two days per week. This model may more closely mimic the natural development of insulin resistance, associated with modest dietary changes along with intermittent curcumin consumption in human subjects that we can expect. We show in this model that curcumin supplementation blocked the effect of HFD on fat gain, improved insulin sensitivity and glucose disposal, and reduced intra-hepatic lipid content. In addition to the confirmation of the effect of curcumin in stimulating anti-oxidative signaling and attenuating inflammatory signaling in hepatocytes, we found that curcumin reduces mRNA levels of ChREBP and SREBP1-c, two key transcription factors for hepatic lipogenesis, as well as L-PK, an important downstream target of ChREBP [31], [37]. However, we did not observe that in mature adipocytes curcumin stimulates Wnt signaling components or Wnt target genes. We therefore suggest that curcumin exerts its beneficial effect in our HFD fed mouse model via attenuating oxidative stress and inflammatory response in the adipose tissue, and reducing lipogenesis in the liver, without the stimulation Wnt activity in mature adipocytes.

It should be noted that the activation of Wnt signaling is strongly associated with the development and progression of colon cancer and other tumors. The chemotherapeutic effect of curcumin has been partially attributed to the repression of Wnt activity [38]. For example, in the LNCaP prostate cancer cells, curcumin represses total β-cat level, as well as GSK-3 phosphorylation, associated with reduced c-Myc and cyclin D1 expression [38]. In addition, a recent study indicated that curcumin disrupts the mammalian target of rapamycine complex (mTOR) [39]. mTOR is not only a downstream target of insulin signaling, but also serves as an effector of the Wnt signaling pathway [27], [40]. Thus, the repressive effect of curcumin on mTOR further supports the notion that curcumin might repress Wnt activity in cancer cells. In the current study, we show that HFD induced hepatic expression of phosphorylated S6K1, a downstream target of mTOR. Curcumin consumption suppressed S6K1 phosphorylation.

The importance of Wnt signaling pathway in metabolic homeostasis has been broadly recognized recently [41]. Wnt10b is abundantly expressed in mesenchymal precursor cells. Wnt10b mediated Wnt activation stimulates the expression of osetogenic genes at the expense of adipogenic genes [18]. Furthermore, ectopic expression of Wnt10b in transgenic mice impairs the development of the adipose tissue and these mice are resistant to HFD induced obesity [42], [43]. Very recently, a study demonstrated that in the 3T3-L1 cell model, the repression of adipogenic differentiation was accompanied by Wnt/β-cat activation [19]. The authors found that during adipocyte differentiation, curcumin reduced the expression of the components of the destructive complex that are responsible for β-cat degradation, including CK1α, GSK-3β and Axin, accompanied by increased expression of total β-cat, Wnt10b, the Wnt pathway receptor Fz2, the co-receptor LRP5, as well as the Wnt targets c-Myc and cyclin D1 [19]. This study provides a potential novel molecular mechanism to explain the repressive effect of curcumin on adipogenesis. In contrast, we found in the current study that the stimulatory effect of curcumin on Wnt signaling does not occur in mature adipocytes. How this plant dietary compound exerts opposite effects on Wnt signaling pathway in pre-adipocytes versus mature adipocytes deserves further investigations. Nevertheless, we have previously noted cell-type specific effects of Wnt and/or insulin signaling. For example, both insulin and lithium chloride, the latter mimics Wnt activation, stimulate proglucagon gene (gcg) transcription in gut endocrine L cells, but repress the same gcg gene in pancreatic islets [44], [45], [46]. Furthermore, we found the stimulatory effect of insulin on β-cat Ser675 phosphorylation in the gut [22], but not in adipocytes (this study). Finally, one study has shown that in skeletal muscle cells, Wnt activation increases insulin sensitivity through reciprocal regulation of Wnt10b and SREBP-1c [47], while another group showed that Wnt activation in mature adipocytes leads to adipocyte dedifferentiation and insulin resistance [20].

In this study, curcumin blocked the effect of HFD on macrophage infiltration in adipose tissue, associated with the repression of NF-κB level and JNK activity, the improvement of insulin stimulated PKB phosphorylation in adipose tissue and liver, as well as glucose disposal. These observations are consistent with current concepts that the activation of endogenous anti-oxidative system and the repression of inflammatory signaling in adipocytes improve insulin resistance [48]. Whether there are additional mechanisms underlying the improvement of insulin signaling by curcumin supplementation deserves further investigations. For example, mTOR is involved in the development of insulin resistance via a negative feedback loop, i.e. the inhibition of IRS-1 tyrosine phosphorylation [49], while the inhibitory effect of curcumin on mTOR has been demonstrated in certain cancer cells [39], [50]. Since Wnt activation in adipocytes may lead to insulin resistance [20], the inhibition of mTOR by curcumin may result in increased insulin sensitization, because mTOR is also among the effectors of the Wnt signaling pathway [27], [40],

Accelerated hepatic lipogenesis is commonly observed in a number of metabolic disorders, including insulin resistance, metabolic syndrome and T2D [51]. The increase in lipogenesis is another mechanism by which HFD consumption leads to obesity and diabetes. ChREBP and SREBP-1c are two key transcription factors for genes that encode lipogenic enzymes [31], [37]. Very little is known about the effect of curcumin on hepatic lipogenesis, although a recent study showed that in adipocytes curcumin inhibits fatty acid synthase (FAS) [52]. We found that curcumin reduced liver weight and intra-hepatic lipid content. More importantly, dietary curcumin was shown to repress SREBP-1c and ChREBP expression. This, along with the repression of L-PK by curcumin and the in vitro ChREBP LUC reporter analysis, suggest that inhibition of ChREBP expression and function are among the mechanisms by which this dietary component prevents obesity and its associated metabolic defects. It should also be pointed out that curcumin can block cardiac hypertrophy and the implicated underlying mechanism was the repression of p300-HAT activity and hence the inhibition of p300 acetylation of certain transcription factors [13]. ChREBP was found to require p300 or CBP as a co-factor in stimulating the expression of TxNIP [33]. We found here that TxNIP protein and mRNA expression in curcumin fed mice was also reduced. It remains to be determined whether p300 inhibition is among the mechanisms by which curcumin reduces the expression of L-PK and other targets of ChREBP.

Together, our observations confirm that curcumin improves insulin signaling, glucose disposal, and blocks obesity during HFD consumption. Our data confirm that in this chronic HFD feeding model, the function of curcumin is mediated via increasing the capability of the animals in anti-oxidative stress and attenuating inflammatory response in adipocytes. Furthermore, in mature adipocytes, this appears occur independent of Wnt activation as curcumin did not activate Wnt pathway components or Wnt downstream target genes. Finally, we revealed the repressive effect of curcumin on hepatic lipogeneis, associated with the inhibition of ChREBP and SREBP-1c expression. Further investigation is required to determine whether the repressive property of curcumin on p300/CBP is additionally involved in reducing hepatic lipogenesis. Thus, the development of curcumin as a therapy for obesity, insulin resistance and T2D is supported.

## Supporting Information

Figure S1 **No detectable defect by HFD and no appreciable improvement by curcumin on insulin stimulated PKA Ser473 phosphorylation in muscles-** The three groups of mice fed with indicated diet for 28 weeks were fasted over night and injected with PBS or insulin. After 30 min, samples of soleus (**A**) and gastrocnemius (**B**) were prepared and immunoblotted with PKB or Ser473 phosphorylated PKB (p-PKB) antibody.
(TIF)

Figure S2 **Curcumin moderately reduced hepatic NF-κB activity in HFD fed mice although our HFD did not cause an appreciable elevation of NF-kB activity-** Samples from liver of the three groups of mice were prepared for Western blotting with indicated antibody.
(TIF)

Figure S3 **No significance difference was observed on hepatic SCD-1 expression in our animal model. RT-PCR were conducted with the following SCD-1 primes.** Forward:5′-CTACAAGCCTGGCCTCCTGC-3′ Reverse:5′-GGCACCCAGGGAAACCAGGA-3′. N = 3 for each group.
(TIF)

## References

[1] Ogden CL, Yanovski SZ, Carroll MD, Flegal KM (2007). The epidemiology of obesity.. Gastroenterology.

[2] Hotamisligil GS (2006). Inflammation and metabolic disorders.. Nature.

[3] Furukawa S, Fujita T, Shimabukuro M, Iwaki M, Yamada Y (2004). Increased oxidative stress in obesity and its impact on metabolic syndrome.. J Clin Invest.

[4] Hoehn KL, Salmon AB, Hohnen-Behrens C, Turner N, Hoy AJ (2009). Insulin resistance is a cellular antioxidant defense mechanism.. Proc Natl Acad Sci U S A.

[5] Dentin R, Benhamed F, Hainault I, Fauveau V, Foufelle F (2006). Liver-specific inhibition of ChREBP improves hepatic steatosis and insulin resistance in ob/ob mice.. Diabetes.

[6] Xu F, Gao Z, Zhang J, Rivera CA, Yin J (2010). Lack of SIRT1 (Mammalian Sirtuin 1) activity leads to liver steatosis in the SIRT1+/- mice: a role of lipid mobilization and inflammation.. Endocrinology.

[7] Alappat L, Awad AB (2010). Curcumin and obesity: evidence and mechanisms.. Nutr Rev.

[8] Yu Z, Shao W, Chiang Y, Foltz W, Zhang Z (2010). Oltipraz upregulates the nuclear respiratory factor 2 alpha subunit (NRF2) antioxidant system and prevents insulin resistance and obesity induced by a high-fat diet in C57BL/6J mice.. Diabetologia.

[9] Bereswill S, Munoz M, Fischer A, Plickert R, Haag LM (2010). Anti-inflammatory effects of resveratrol, curcumin and simvastatin in acute small intestinal inflammation.. PLoS One.

[10] Mukhopadhyay A, Banerjee S, Stafford LJ, Xia C, Liu M (2002). Curcumin-induced suppression of cell proliferation correlates with down-regulation of cyclin D1 expression and CDK4-mediated retinoblastoma protein phosphorylation.. Oncogene.

[11] Jaiswal AS, Marlow BP, Gupta N, Narayan S (2002). Beta-catenin-mediated transactivation and cell-cell adhesion pathways are important in curcumin (diferuylmethane)-induced growth arrest and apoptosis in colon cancer cells.. Oncogene.

[12] Weisberg SP, Leibel R, Tortoriello DV (2008). Dietary curcumin significantly improves obesity-associated inflammation and diabetes in mouse models of diabesity.. Endocrinology.

[13] Morimoto T, Sunagawa Y, Kawamura T, Takaya T, Wada H (2008). The dietary compound curcumin inhibits p300 histone acetyltransferase activity and prevents heart failure in rats.. J Clin Invest.

[14] Barnes PJ (2009). Role of HDAC2 in the pathophysiology of COPD.. Annu Rev Physiol.

[15] Jin T (2008). The WNT signalling pathway and diabetes mellitus.. Diabetologia.

[16] Manolagas SC, Almeida M (2007). Gone with the Wnts: beta-catenin, T-cell factor, forkhead box O, and oxidative stress in age-dependent diseases of bone, lipid, and glucose metabolism.. Mol Endocrinol.

[17] Schinner S (2009). Wnt-signalling and the metabolic syndrome.. Horm Metab Res.

[18] Krishnan V, Bryant HU, Macdougald OA (2006). Regulation of bone mass by Wnt signaling.. J Clin Invest.

[19] Ahn J, Lee H, Kim S, Ha T (2010). Curcumin-induced suppression of adipogenic differentiation is accompanied by activation of Wnt/beta-catenin signaling.. Am J Physiol Cell Physiol.

[20] Gustafson B, Smith U (2010). Activation of canonical wingless-type MMTV integration site family (Wnt) signaling in mature adipocytes increases beta-catenin levels and leads to cell dedifferentiation and insulin resistance.. J Biol Chem.

[21] Oakes ND, Thalen PG, Jacinto SM, Ljung B (2001). Thiazolidinediones increase plasma-adipose tissue FFA exchange capacity and enhance insulin-mediated control of systemic FFA availability.. Diabetes.

[22] Sun J, Khalid S, Rozakis-Adcock M, Fantus IG, Jin T (2009). P-21-activated protein kinase-1 functions as a linker between insulin and Wnt signaling pathways in the intestine.. Oncogene.

[23] Sirek AS, Liu L, Naples M, Adeli K, Ng DS (2009). Insulin stimulates the expression of carbohydrate response element binding protein (ChREBP) by attenuating the repressive effect of Pit-1, Oct-1/Oct-2, and Unc-86 homeodomain protein octamer transcription factor-1.. Endocrinology.

[24] Jin T, Drucker DJ (1996). Activation of proglucagon gene transcription through a novel promoter element by the caudal-related homeodomain protein cdx-2/3.. Mol Cell Biol.

[25] Tirosh A, Potashnik R, Bashan N, Rudich A (1999). Oxidative stress disrupts insulin-induced cellular redistribution of insulin receptor substrate-1 and phosphatidylinositol 3-kinase in 3T3-L1 adipocytes. A putative cellular mechanism for impaired protein kinase B activation and GLUT4 translocation.. J Biol Chem.

[26] Taurin S, Sandbo N, Qin Y, Browning D, Dulin NO (2006). Phosphorylation of beta-catenin by cyclic AMP-dependent protein kinase.. J Biol Chem.

[27] Jin T, George Fantus I, Sun J (2008). Wnt and beyond Wnt: multiple mechanisms control the transcriptional property of beta-catenin.. Cell Signal.

[28] Sun J, Jin T (2008). Both Wnt and mTOR signaling pathways are involved in insulin-stimulated proto-oncogene expression in intestinal cells.. Cell Signal.

[29] Monteiro R, Azevedo I (2010). Chronic inflammation in obesity and the metabolic syndrome.. Mediators Inflamm 2010.

[30] Nishiyama T, Mae T, Kishida H, Tsukagawa M, Mimaki Y (2005). Curcuminoids and sesquiterpenoids in turmeric (Curcuma longa L.) suppress an increase in blood glucose level in type 2 diabetic KK-Ay mice.. J Agric Food Chem.

[31] Uyeda K, Repa JJ (2006). Carbohydrate response element binding protein, ChREBP, a transcription factor coupling hepatic glucose utilization and lipid synthesis.. Cell Metab.

[32] Yu FX, Chai TF, He H, Hagen T, Luo Y (2010). Thioredoxin-interacting protein (Txnip) gene expression: sensing oxidative phosphorylation status and glycolytic rate.. J Biol Chem.

[33] Cha-Molstad H, Saxena G, Chen J, Shalev A (2009). Glucose-stimulated expression of Txnip is mediated by carbohydrate response element-binding protein, p300, and histone H4 acetylation in pancreatic beta cells.. J Biol Chem.

[34] Hatcher H, Planalp R, Cho J, Torti FM, Torti SV (2008). Curcumin: from ancient medicine to current clinical trials.. Cell Mol Life Sci.

[35] Ejaz A, Wu D, Kwan P, Meydani M (2009). Curcumin inhibits adipogenesis in 3T3-L1 adipocytes and angiogenesis and obesity in C57/BL mice.. J Nutr.

[36] Lee YK, Lee WS, Hwang JT, Kwon DY, Surh YJ (2009). Curcumin exerts antidifferentiation effect through AMPKalpha-PPAR-gamma in 3T3-L1 adipocytes and antiproliferatory effect through AMPKalpha-COX-2 in cancer cells.. J Agric Food Chem.

[37] Postic C, Dentin R, Denechaud PD, Girard J (2007). ChREBP, a transcriptional regulator of glucose and lipid metabolism.. Annu Rev Nutr.

[38] Choi HY, Lim JE, Hong JH (2010). Curcumin interrupts the interaction between the androgen receptor and Wnt/beta-catenin signaling pathway in LNCaP prostate cancer cells.. Prostate Cancer Prostatic Dis.

[39] Beevers CS, Chen L, Liu L, Luo Y, Webster NJ (2009). Curcumin disrupts the Mammalian target of rapamycin-raptor complex.. Cancer Res.

[40] Inoki K, Ouyang H, Zhu T, Lindvall C, Wang Y (2006). TSC2 integrates Wnt and energy signals via a coordinated phosphorylation by AMPK and GSK3 to regulate cell growth.. Cell.

[41] Jin T, Liu L (2008). The Wnt signaling pathway effector TCF7L2 and type 2 diabetes mellitus.. Mol Endocrinol.

[42] Longo KA, Wright WS, Kang S, Gerin I, Chiang SH (2004). Wnt10b inhibits development of white and brown adipose tissues.. J Biol Chem.

[43] Huang X, Charbeneau RA, Fu Y, Kaur K, Gerin I (2008). Resistance to diet-induced obesity and improved insulin sensitivity in mice with a regulator of G protein signaling-insensitive G184S Gnai2 allele.. Diabetes.

[44] Yi F, Sun J, Lim GE, Fantus IG, Brubaker PL (2008). Cross talk between the insulin and Wnt signaling pathways: evidence from intestinal endocrine L cells.. Endocrinology.

[45] Yi F, Brubaker PL, Jin T (2005). TCF-4 mediates cell type-specific regulation of proglucagon gene expression by beta-catenin and glycogen synthase kinase-3beta.. J Biol Chem.

[46] Philippe J (1989). Glucagon gene transcription is negatively regulated by insulin in a hamster islet cell line.. J Clin Invest.

[47] Abiola M, Favier M, Christodoulou-Vafeiadou E, Pichard AL, Martelly I (2009). Activation of Wnt/beta-catenin signaling increases insulin sensitivity through a reciprocal regulation of Wnt10b and SREBP-1c in skeletal muscle cells.. PLoS One.

[48] Houstis N, Rosen ED, Lander ES (2006). Reactive oxygen species have a causal role in multiple forms of insulin resistance.. Nature.

[49] Shah OJ, Wang Z, Hunter T (2004). Inappropriate activation of the TSC/Rheb/mTOR/S6K cassette induces IRS1/2 depletion, insulin resistance, and cell survival deficiencies.. Curr Biol.

[50] Rafiee P, Binion DG, Wellner M, Behmaram B, Floer M (2010). Modulatory effect of curcumin on survival of irradiated human intestinal microvascular endothelial cells: role of Akt/mTOR and NF-{kappa}B.. Am J Physiol Gastrointest Liver Physiol.

[51] Denechaud PD, Dentin R, Girard J, Postic C (2008). Role of ChREBP in hepatic steatosis and insulin resistance.. FEBS Lett.

[52] Zhao J, Sun XB, Ye F, Tian WX (2011). Suppression of fatty acid synthase, differentiation and lipid accumulation in adipocytes by curcumin.. Mol Cell Biochem.

