# Supplementary figures and images for: Curcumin Prevents High Fat Diet Induced Insulin Resistance and Obesity via Attenuating Lipogenesis in Liver and Inflammatory Pathway in Adipocytes
Source: PLoS One. 2012 Jan 9;7(1):e28784. doi: 10.1371/journal.pone.0028784 (PMC3253779; doi:10.1371/journal.pone.0028784)

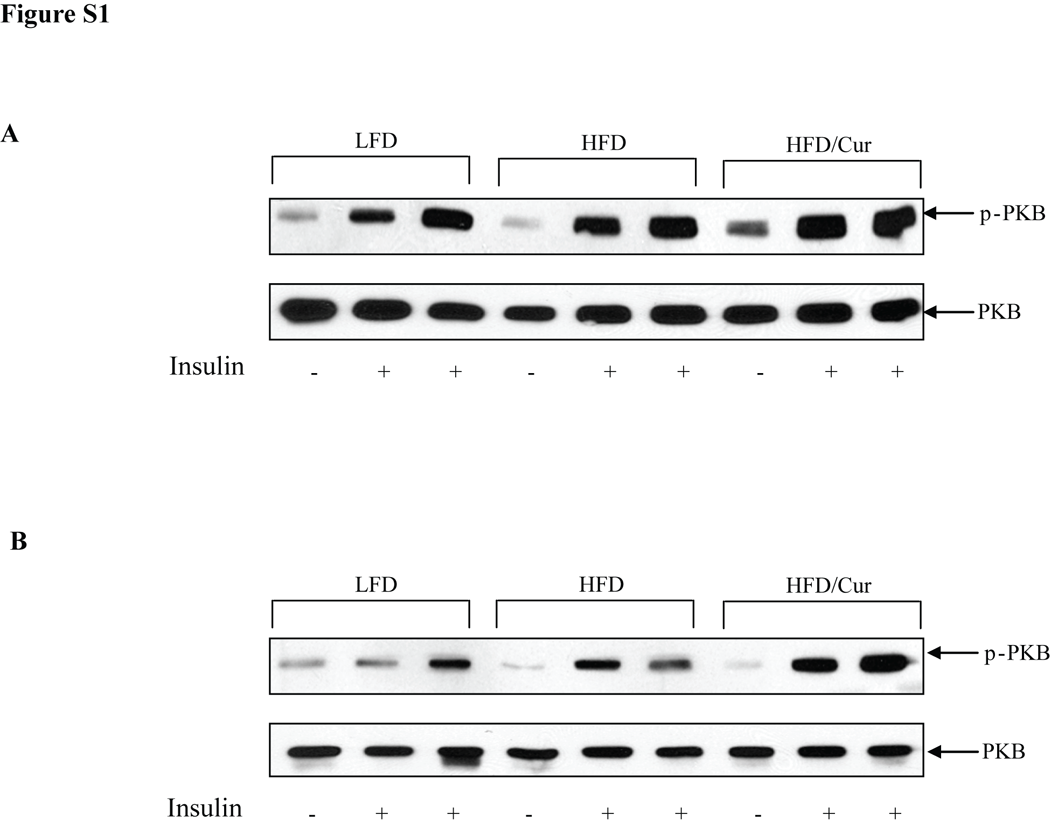

Supplement: Figure S1 — No detectable defect by HFD and no appreciable improvement by curcumin on insulin stimulated PKA Ser473 phosphorylation in muscles- The three groups of mice fed with indicated diet for 28 weeks were fasted over night and injected with PBS or insulin. After 30 min, samples of soleus (A) and gastrocnemius (B) were prepared and immunoblotted with PKB or Ser473 phosphorylated PKB (p-PKB) antibody. (TIF) [file pone.0028784.s001.tif]

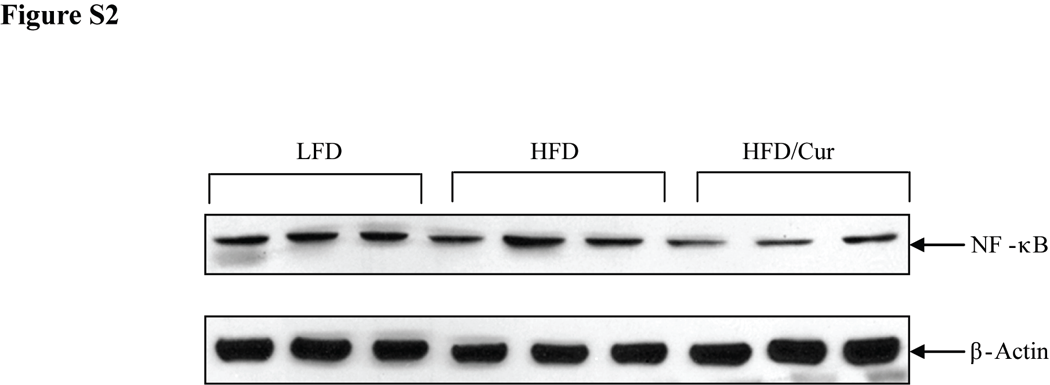

Supplement: Figure S2 — Curcumin moderately reduced hepatic NF-κB activity in HFD fed mice although our HFD did not cause an appreciable elevation of NF-kB activity- Samples from liver of the three groups of mice were prepared for Western blotting with indicated antibody. (TIF) [file pone.0028784.s002.tif]

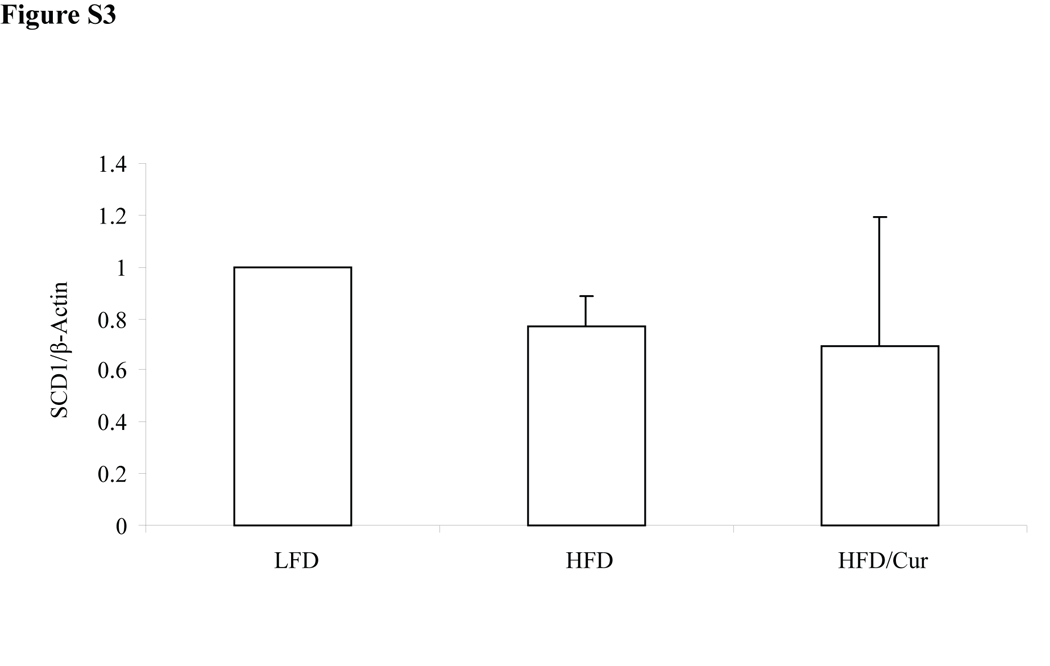

Supplement: Figure S3 — No significance difference was observed on hepatic SCD-1 expression in our animal model. RT-PCR were conducted with the following SCD-1 primes. Forward:5′-CTACAAGCCTGGCCTCCTGC-3′ Reverse:5′-GGCACCCAGGGAAACCAGGA-3′. N = 3 for each group. (TIF) [file pone.0028784.s003.tif]
